# *Persuasion or coercion?* An empirical ethics analysis about the use of influence strategies in mental health community care

**DOI:** 10.1186/s12913-022-08555-5

**Published:** 2022-10-21

**Authors:** Emanuele Valenti, Domenico Giacco

**Affiliations:** 1 Centre for Ethics in Medicine, Bristol Medical School, Population Health School Sciences, University of Bristol, Canynge Hall, Bristol, BS8 2 PS UK; 2 Department of Mental Health and Wellbeing, Warwick Medical School, University of Warwick, Coventry, CV 7 AL UK

**Keywords:** Coercion, Decision-making, Influence, Leverage, Pressures, Autonomy

## Abstract

**Background:**

Influence strategies such as persuasion and interpersonal leverage are used in mental health care to influence patient behaviour and improve treatment adherence. One ethical concern about using such strategies is that they may constitute coercive behaviour ("informal coercion") and negatively impact patient satisfaction and the quality of care. However, some influence strategies may affect patients' perceptions, so an umbrella definition of “informal coercion” may be unsatisfactory. Furthermore, previous research indicates that professionals also perceive dissonance between theoretical explanations of informal coercion and their behaviours in clinical practice. This study analysed mental health professionals’ (MHPs) views and the perceived ethical implications of influence strategies in community care.

**Methods:**

Qualitative secondary data analysis of a focus group study was used to explore the conflict between theoretical definitions and MHPs’ experiences concerning the coerciveness of influence strategies. Thirty-six focus groups were conducted in the main study, with 227 MHPs from nine countries participating.

**Results:**

The findings indicate that not all the influence strategies discussed with participants can be defined as “informal coercion”, but they become coercive when they imply the use of a lever, have the format of a conditional offer and when the therapeutic proposal is not a patient’s free choice but is driven by professionals. MHPs are rarely aware of these tensions within their everyday practice; consequently, it is possible that coercive practices are inadvertently being used, with no standard regarding their application. Our findings suggest that levers and the type of leverage used in communications with the patient are also relevant to differentiating leveraged and non-leveraged influence.

**Conclusion:**

Our findings may help mental health professionals working in community care to identify and discuss influence strategies that may lead to unintended coercive practices.

## Background

Article 12 of the UN Convention on the Right of Persons with Disabilities (CRPD) [1] extended the concept of disability to all people who have a physical, mental, cognitive or sensory impairment that may reduce their opportunities for social inclusion and respect for their equal rights. By preserving legal capacity, the CRPD obliges that the rights, will and preferences of people with disabilities are respected. However, there are suggestions that mental health care professionals (MHPs) struggle to implement fully collaborative approaches when working with community patients [2–9].

In psychiatric practice, strategies are often used to influence patient decisions. For example, MHPs can aim, in the perceived best interests of the patient, to influence patients’ decisions to increase treatment compliance and avoid disengagement from services [10–12]. The need to clarify the nature of influences is not only a concern in the mental health literature. Still, it has also become a matter of interest in both critical care [13]and paediatrics [14, 15]. In Europe, studies have explored how influence determines patients’ perceptions [16, 17] and MHPs’ views [18, 19].

The theoretical body developed to define influences on community mental health has focused on the inquiry of informal (or covert) coercion. This has caused discomfort among MHPs who consider coercion ethically wrong [20]. No theoretical distinctions have been made between “persuasive” and “coercive” influence strategies [12], i.e. persuasion and coercion. Nevertheless, empirical research in mental health has identified four different types of influence strategies: (1) persuasion (i.e. convincing the patient through rational arguments); (2) interpersonal leverage (i.e. using a patient’s emotional dependence on family, carers or mental health care professionals); (3) inducement (i.e. offering incentives such housing, money or work); and (4) threat (i.e. withholding or denying some benefit, or threatening patients with involuntary hospitalisation [21]. Although the relationship between each of these influence strategies has been frequently explained as a *continuum* from persuasion to threat and then to other forms of formal coercion (i.e. compulsory treatment and involuntary hospitalisation) [2–4, 6, 11, 21–23], research findings indicate substantial differences among these strategies. For instance, it was found that the impact of persuasion on patient perceptions of care differs significantly from that of coercion [24–26]. Persuasion and inducement can be regarded as “positive pressures” because they preserve a patient’s choice, in contrast to threat and formal coercion, which reduce patients’ options and limit courses of action [25, 27, 28]. MHPs’ perceptions about the use of influence in community mental health care are often polarised, and there can be dissonances between personal beliefs and behaviours in clinical practice [29]. The coercive nature of leveraged influence strategies can be under- or overestimated by mental health practitioners [3, 30, 31]. Consequently, informal coercion can be used unintentionally [32] or not used when required [33]. Of the existing influence strategies identified in the literature, the threat is the only undisputedly considered coercive [21].

We implemented a secondary analysis of empirical data to explore the nature and variety of influence strategies in community mental health care settings and their ethical significance, being focused on why definitions of pressures and leverages provided during the discussion were retained by MHPs as coercion or non-coercion. Empirical bioethics aims to interrogate normative questions by exploring the observed data [34]. Bioethicists deem that empirical research helps enrich theoretical constructs and contributes to understanding ethical issues related to practice and behaviours. In this study, we sought to understand the ethical implications of the tension between the conceptualisation of pressures and leverages proposed by literature [21] and participants’ clinical experiences of influence strategies. To this end, the views of MHPs working in different countries were canvassed.

Our thematic analysis addressed the following research question: what are the boundaries between non-coercion and coercion when using influence in community care? To answer this question, we discussed different strategies used by MHPs to influence the behaviour of their patients. We explored their views as to whether and when these strategies are merely “persuasive”, or they can be labelled as informal coercion.

## Methods

### Study design

This study was a qualitative secondary thematic analysis of data from a previous focus group study, which has been described in detail elsewhere [28]. The primary study was an inductive thematic analysis exploring the attitudes and experiences of informal coercion of MHPs from different psychiatric traditions and backgrounds. Because of the differences in mental health services among countries, Valenti et al. [28] used “psychiatry” as an umbrella term to describe a service provided by various professionals, including nurses, social workers, clinical psychologists and occupational therapists. The present secondary analysis is a supplementary inquiry of a question emerging from more recent investigations [30–32]. To answer the new research question, “reflexive balancing” [35] has been applied to frame the data. This method is beneficial when ethical theory or principles deal with a conflicting experience or practice. The present secondary thematic analysis explored the possibility of finding a balanced solution between normative issues and clinical practice.

### Population

The study was conducted across different sites and countries, exploring various cultural contexts and practices. The sample included professionals from nine countries: Canada, Mexico, Chile, Croatia, the UK, Norway, Sweden, Italy and Spain. Germany was included in the primary study but excluded from the secondary analysis because of the lack of a German-speaking analyst. Due to the lack of community services in some of the countries included in the study (i.e. Italy, Spain, Mexico, Chile and Croatia), participants were recruited from hospital psychiatric units and community services where available. Inclusion criteria were age between 18 and 65 years, at least 1 year of experience as an MHP and experience working whit outpatients with severe mental illness. Purposive sampling considered gender balance and the representation of all professional roles involved in the care of mental health outpatients, including psychiatrists, mental health nurses, clinical psychologists, occupational therapists, social workers and case managers, among others (see Table 1).

**Table 1:** Participants’ socio-demographic characters

	^***Psychiatrist***^	^**Nurse**^	^**Clinical psychologist**^	^**Social worker**^	^**Occupational therapist**^	^**Other mental healthcare professionals**^	
*COUNTRY*	*Participants (N)*						
**Canada**	2	10	0	5	0	3 (case workers)	20
**Croacia**	15	3				1 (medical technician)	19
**United Kingdom**	2	10	2	8	2	1 (pharmacist)	26
**Norway**	5	10	12	1	1	1 (special needs educator)	30
**Sweden**	4	7	0	8	0	0	19
**Italy**	16	12	4	1	0	0	33
**Spain**	10	10	4	4	1	1 (general practitioner)	30
**Mexico**	3	0	16	2	0	3 (1 medical nutritionist, 1 medical technician, 1 unknown)	24
**Chile**	12	5	6	3	0	0	26
**Total**	69	67	44	27	4	11	227

The study was designed to explore patterns and themes across different countries and mental health services. Following the rule of thumb suggested by Krueger and Casey [36], we conducted four focus groups in each country to ensure theoretical saturation. Focus group participants were recruited from each country to discuss the same standard of care for each mental health care system. After all four influence strategies represented by case vignettes had been commented on and appropriately debated by participants in each of the focus groups, data saturation was reached.

### Procedures

The primary study had a core group of researchers at the Unit for Social and Community Psychiatry, Queen Mary University of London and researchers in each country. Secondary data analysis was performed by a bioethicist at the Centre for Ethics in Medicine, University of Bristol and by a clinical and academic psychiatrist at Division of Health Sciences, Warwick Medical School. Researchers with experience in facilitating focus groups moderated the discussion in each country. The same facilitator in each country moderated all the national focus groups and was supported by an observer taking notes to simplify the transcription process and record all non-verbal communication aspects considered relevant for transcript analysis. The facilitators had different bioethics, psychology and psychiatry backgrounds and followed a structured process described in the topic guide [29].

At the beginning of the focus group session, participants were provided with a brief introduction about the facilitator’s background and the research project, as well as instructions for participating in the focus group. After the first question, oriented to collect sociodemographic characteristics, the facilitator provided and elucidated a definition of pressures and leverages proposed by literature [21]. Personal experiences and views were elicited using a set of case vignettes [22] describing different influence strategies (Table 2). The facilitator alternated between specific structured questions in the topic guide and Socratic questioning to help professionals disclose their experiences and views. The duration of the focus groups varied between 60 and 120 min, and discussions were digitally recorded and transcribed verbatim. Focus groups in the UK, Canada, Chile, Italy, Mexico and Spain were conducted, transcribed and analysed in the original languages; focus groups in Croatia, Sweden and Norway were conducted and transcribed in their official language, with the transcripts subsequently translated into English at the Unit for Social and Community Psychiatry. However, for the thematic analysis, all transcripts were in the languages spoken by the core team members.

**Table 2:** Case vignettes

Case Vignette 1
**The patient is a 30-year-old woman with bipolar disorder who has had several admissions to the hospital over the years, often as involuntary hospitalisation. Between hospital treatments, she keeps well and functions as long as she accepts medication and support. Without these, she quickly becomes unwell.**
Persuasion
*The clinician in the out-patient service is increasingly concerned about the situation and keen to try and avert another damaging relapse. The clinician talks to the patient and explains the evidence for medication in bipolar disorder and the fact that her pattern of relapse indicates that this applies to her.*
Interpersonal leverage
*The clinician tries to appeal to the patient because they have known each other for a long time; he has always been there to help and would not advise her to do something that was not in her best interests.*
Inducement
*The appeals did not work, and the patient is starting to show early signs of deterioration. There is a sale of children’s clothes coming up, and the patient wants to buy something to give to her daughters when she next sees them. The clinician offers to give her a lift but says he can only do so if she is reasonably well. Whether or not the clinician means to imply she needs to take treatment to gain his assistance is left unclear, but that is the patient’s assumption.*
Threat
*The following week the patient is due to see her daughters. She is still refusing treatment and now shows signs of irritability, which for her, is an early sign of relapse. The clinician explains that the access visit might have to be cancelled if she gets any more irritable or is still refusing treatment and that he must let social services know about the situation.*
Case Vignette 2
***The patient is a 40-year-old man with chronic schizophrenia. He lives alone in a flat with practically no social contact, and he tends to self-neglect. He hears voices and believes the neighbours are spying on him, which makes him very distressed. In the past, he has shown marked improvement when on medication. He has never harmed himself or others. He is willing to see the staff of the community mental health team, but not to take medication or leave the flat to participate in activities.***
Persuasion
*The clinician in the community team who has known the patient for a long time is concerned about the situation and keen to try and reduce the patient’s distress. The clinician talks to the patient and explains the importance of taking medication and engaging in social activities, emphasizing that further refusal of treatment may lead to continued or increased distress and impaired quality of life.*
Interpersonal leverage
*The clinician has repeatedly helped to prevent the patient from being evicted from his flat despite the obvious neglect and inconsistent rent payments. The clinician now says that it is frustrating to continue providing care to the patient unless the patient shows more engagement with treatment.*
Inducement
*The patient is keen on getting a new TV set, but can only afford it if social welfare provides the funding, which requires an application that needs to be supported by the community team. The clinician brings this up and promises to help with such an application if the patient shows more engagement with treatment.*
Threat
*The patient has received another letter from the landlord with the intention to evict him from the flat. The clinician declares that the team will only help the patient to avoid eviction again if he takes medication and/or regularly attends a drop-in centre for some structured activity and social contact.*

The primary study was approved by five national research ethics committees: for the entire research project in Spain, Comite Etico de Investigacion Cientifica, Hospital Universitario Fundacion Alcorcon, (HUFA-CEIC 12/66) and for the collection and analysis of national data in the United Kingdom, Research Ethics Committee, Queen Mary University of London (QMREC2012/80), in Canada, Research Ethics Board, Ontario Shores Centre for Mental Health (REB#13–011-B), in Chile, Hospital Clınico de la Universidad de Chile (63/14–11-2013) and in Sweden, Regionala Etikpro¨vningsna¨mnden Uppsala (DNR 2013/011). The remaining countries did not require separate study approval. All participants provided written informed consent. Because of the compatibility of aims between the primary and secondary analyses, the latter did not require new research ethics committee approval.

### Data analysis

The present analysis examined the participants’ perceptions of the definition of leverages and pressures proposed in the case vignettes and the reasons for their agreement or disagreement to consider them informal coercion. This emerged as a post hoc matter of interest due to the new literature and concepts that have become available over time [31–33]. The analysis explored the entire dataset of the primary study, except for transcripts from the German focus groups. All transcripts were imported into the qualitative software package QSR NVivo 12. A coding frame was designed deductively through a search query structure within the software. The analysis focused on identifying participants’ views on each of the four treatment pressures described in Szmulker and Appelbaum [21]. The four main themes identified represent a synthesis of all the arguments provided by participants to define each option introduced as “coercion” or “non-coercion”. One researcher (EV) performed open coding on 18 transcripts to generate the initial codes. A second researcher (DG) developed a preliminary coding framework, assessed its reliability and coded another 18 transcripts (two per country). The codes were compared against all 18 transcripts coded previously by the first researcher (EV), and rates of agreement ranged from 80% to 98.5%. Minor changes were made to the coding, and the final coding framework was used to complete the coding of all transcripts.

Secondary analysis was carried out by an academic bioethicist (EV) and one clinical academic psychiatrist (DG). A “top-down” thematic analysis [37] was performed to drive the research through a comparative reading of theory and experience. The investigation was conducted using an iterative process, splitting and splicing codes, to identify themes describing the main characteristics of the proposed spectrum of pressures. Through the visualisation functions included in NVivo, a conceptual model was created to sketch the research hypothesis and classify nodes deductively with regard to the four different influence strategies and their descriptions as coercive or non-coercive. Connections between nodes from selected codes were explored visually, with the charts converted into logic models. Two researchers (EV, DG) discussed and fine-tuned the model, reaching a consensus about the relationship between the data and conclusions. The secondary analysis was supplementary and focused on emergent issues not addressed in the primary study (i.e. exploring the normative issues implicit in clinical practice). All the problems related to secondary data analysis [38] have been mitigated. The data collected for the primary purpose fit the purpose of the secondary analysis. One of the researchers (EV) was involved in both the primary study and secondary analysis, and the data were verified through a triangulation process, as described above. One researcher (EV) involved in both investigations addressed legal and ethical issues related to consent and confidentiality.

## Results

MHPs working in nine countries were involved in this study. Four focus groups were conducted in each country (36 focus groups in total). In all, 227 MHPs (152 women; 67.0%) were included in the focus groups, with between 4 and 13 participants attending each group (mean 6.2). As reported in Table 1, the groups included psychiatrists (*n* = 69), nurses (*n* = 67), clinical psychologists (*n* = 44), social workers (*n* = 27), occupational therapists (*n* = 4) and others (*n* = 11).

### Themes

MHPs expressed their agreement/disagreement with the labelling of influence strategies described in the case vignettes as “informal coercion”. Four main themes were identified to explore the relationship between the definition provided [21] and participants’ views. Namely, persuasion (Table 3), interpersonal leverage (Table 4), inducement (Table 5) and threat (Table 6). Within each of these themes, the experiences and views of the participating MHPs were compared.

**Table 3:** Persuasion

Theme 1 Persuasion			
*Perceptions*	*Sub theme*	*Excerpt*	*Quotes*
**Non-Coercion**	Professional Information	E.1	(NO208, nurse) *No, I don’t agree with that* (it refers to the case vignette presenting persuasion as coercion) *[…] I believe they should get the information from us then that they get it from somewhere else, but that we can then go in and explain and provide good information and meet them with the concerns they have*
	Assessment	E.2	*(CH315 psychiatrist) (the patient) perceived it was his own decision. The argument I used has been essentially the confidence, I mean, it’s like to say the doctor doesn’t lay me, but she just tells me about something can harm me […] of course, this is persuasion*
	Motivation	E.3	(CA404, psychiatrist) *So that’s kind of persuasive, it’s not saying: “If you cannot do this, then you have to do that.” It’s saying, “These are what your choices are,” but you show whatever you want for the patient, or whatever they want, you say to them “If you want this, then you’ve got to do that.” […] I don’t think we typically consider that persuasion* (he refers to persuasion as presented in case-vignette as coercion)*, as much as… encouragement, offering hope*
**Coercion**	Informational manipulation	E.4	(CR208, psychiatrist) *Withholding information. In the example, a patient has psychotic symptoms, and he doesn’t perceive them, but rather reports only depressive symptoms. And then I tell him for a cure that has antipsychotic as well as anti-depressive activity. I underline this anti-depressive part to persuade him to take the care and keep this*
	Deceit	E.5	(IT103, psychiatrist) *because I could say persuasion has an enormous grading of options. If I’m a good persuader, I can deceive a patient in a few seconds. In that sense persuasion became a kind of psychological violence If I have the skills because the simple information is poor communication, there’s not the option to propose a specific choice to the patient: information is neutral, persuasion is taking you away in your best interests*
	Manipulation	E.6	(MX104, psychiatrist) *you are hooking the patient […] you are using alternatives to mislead and hook the patient […] it means manipulation but at the unconscious level*
	Threat	E.7	(EN316, social worker) *You know, the more unwell they are, the more likely you’re going to use [murmurs of agreement, indecipherable talking over each other] the bigger, more questionable form of persuasion, “Do this or, you know…” in the hope that they get some insight and their mental health stabilises*
	Conditional offer	E.8	(SW416, psychiatrist) *Then I use persuasion. I say things like: “I think you get worse if you don’t take your medicine, and if you don’t take your medicine, you know how it can end up.” My intention is not to threaten them, but to remind them*

**Table 4:** Interpersonal leverage

Theme 2 Interpersonal leverage			
*Perceptions*	*Sub theme*	*Excerpt*	*Quotes*
**Non-coercion**	Social network involvement	E.9	(EN105, nurse) *Or you can, if you can’t persuade them you get someone that knows them better, a family member or someone to persuade them, or a friend, or their care coordinator who knows them better than we do. You’re trying to get. You work with that first. You don’t jump straight to the threat*
	Handling emotion	E.10	(MX106, psychiatrist) *if you are handling the patient’s emotions intending to promote his/her well-being, and not to obtain a personal benefit […] it is always in the patient’s name*
	Respectful reciprocity	E.11	(CH312, psychiatrist) *persuasion and interpersonal leverage are two strategies frequently used and called in the first case psychoeducation and the second one therapeutic bond, it means the patient should know he/she is having a mutual respect relationship in his/her best interests and never oriented to harming him, the therapeutic bond is frequently used*
	Confidence	E.12	(SP206, psychiatrist*) the second step consists in the explanation about the treatment supported by the patient’s confidence on his/her doctor, because he/she needs this, some influence at a specific moment*
**Coercion**	Leverage on confidence	E.13	(IT216, psychiatrist) *I think interpersonal leverage is coercion […] patient sometimes can establish a trustful relationship with the doctor and the doctor plays, we lever on confidence to convince the patient to change his/her opinion, and this is coercion*
	Unprofessional	E.14	(CA203, nurse) *I don’t think the interpersonal one is acceptable—*(CA201, nurse) *I think that’s unprofessional to say that* (referring to the case vignette showing interpersonal leverage)
	Wrong	E.15	(SW103, social worker) *My spontaneous reaction is that it felt wrong to use this*

**Table 5:** Inducement

Theme 3 Inducement			
*Perception*	*Subtheme*	*Excerpt*	*Quotes*
**Non coercion**	Positive reinforcement	E.16	(EN103, psychiatrist) *The last two blur into each other a little bit, because inducement and threat, like “if you do this, then you can have left, if you don’t do this then we’re gonna take your leave away”, whether that’s an inducement or that’s a threat like it’s a bit like positive reinforcement versus negative reinforcement, but there’s a sliding scale there, I think*
	Negotiation	E.17	(SP102, psychologist) *in the inducement a professional try to convince the patient to take the medication without to use the force, but at the same time trying to negotiate with him, “look, if you accept the treatment you will get this one, or you won’t lose that one (and I give this one)*
	Therapeutic agreement	E.18	(MX212, psychologist) *I would be inclined to the induction in order to improve the patient awareness about his/her circumstances, and with this, would explore any family support in order to assure patient’s compliance […] generally I start with induction in order to inform the patient about his/her circumstances, who will support the patient, which resources will be available to the patient and doctor and see what will manage the therapeutic strategy*
**Coercion**	Enticement	E.19	(SW105, nurse) Cigarettes were mentioned too. Something we can entice them with. Many have cigarettes and they have a cigarette allowance. This is something that we regulate so it’s a question of when they get them or when they don't get them. They have cigarettes that we set aside for them.”If you take your medicine, I'll go and get you a cigarette.” That's how it is
	Coercion	E.20	(IT317, psychiatrist) *perhaps for induction and threat yes (he refers to the definition of coercion), I mean […] you are thinking about you are doing in their best interests. However, I feel induction and threat as coercion*
	Manipulation	E.21	(*CA1 Facilitator) They call it an inducement, or an incentive—(CA101, psychiatrist) I’d call a manipulation, and it’s unwise. And I would object to the last section (it refers to the case vignette 1), where it said, ‘It appears there may be some other agency or institution involved regarding seeing your children.’ So, one would want to know what that is to what you can call anything around access to the children’ coercion’ or again safety, concerning the law*
	Harm doctor-patient relationship	E.22	*(CH309, nurse) I do not agree to use the induction because it can affect the boundary between doctor and patient in the sense that patient is generally demanding. If a patient accedes to the treatment in a change of something, the treatment can fail because the patient will be excessively demanding*
	Extortion in the patient’s best interests	E.23	(*CR301, psychiatrist) (referring to inducement) You call it offering rewards, and I suppose that’s what it could be called […] Now, whether that’s a threat, a reward or extortion… It’s good either way*

**Table 6:** Threat

Theme 4 Threat			
*Perception*	*Sub theme*	*Excerpt*	*Quotes*
**Non-coercion**	Reality assessment	E24	*(IT426, psychiatrist) It is a way to put on her attention the situation because if I tell she cannot see her daughter if she became clinically unbalanced, it could be a threat, but is at the same time a reality assessment, and it’s better to know it in advance in the context of the community service supported by a psychiatrist than to leave the patient directly facing the consequences of his/her clinical circumstance*
	Factual Information	E25	(NO208, nurse) *When you say threat, I feel that this is not what I do. It is more focusing on the facts. However, I know it is not certain that she understands what I say. However, I […] And then I am thinking in such a way that it is not about giving a warning, in my opinion, it is about talking about what is happening when you become unwell*
	Actual threat or anticipation	E26	(CR418, psychiatrist) *And also, the term threat. Different situations can be described with the same term, meaning it can be an actual threat or merely having someone face consequences which are very real. That (referring to case vignette 2) can sound like a threat, but it is a fact that, if a patient makes noise, the police will come*
	Risk/benefit balance	E27	CH111, psychologist) *I do not see this as a threat, what we are doing is to show the patient the consequences of he/she do not take the medication, we do not say to the patient “you did not take the medication. Then I will ask the intervention of the social services or the court. It is like “look, this is happening, can happen this one or that one, or your family can make this decision”*
**Coercion**	Communication style	E28	*(FG209, psychiatrist) Threat is used in the day life practice, but it is a mean of expression than an action with the function to threat […] it is a therapeutic strategy to improve your health without your consent*
	Highest level of coercion	E29	(EN317, nurse) *You know, from a medical point of view and the medical model treatment of a patient with mental health issues, I do not see that happening, because it would mean resorting to the highest level of covert coercion which would be a threat if you have to inform them of the side effects*
	Unethical	E30	(MX313, psychologist) *the threatening someone today is not easy, because there are mechanisms of ethical control in the medical practice, and a doctor is at risk to lose their licence if this happens*
	Untrustworthy	E31	(CA201, nurse): *And I think if you put it out there, ‘if you do not take your meds, you are going to get evicted,’ that does not happen because you are of course advocating for them to keep their housing, then the trust starts to fall apart. Because then you’re threatening things that you are not going to follow through on. Because you want what is in the client’s best interest. So, I think then distrust comes into the relationship, and the relationship is starting to unravel or go the other way- (CA201 Facilitator) And is that something that is a concern to you? (CA201) Trust? Absolutely. Trust is a much more powerful and long-lasting tool than a short-term threat*

### Persuasion: information provision and MHPs’ behaviours and aims

In Table 3, quotes illustrate persuasion as a common influence strategy used in the user–provider encounter to change a patient’s attitude by providing information about clinical decision-making. However, persuasion in community psychiatry can be controversial, and MHPs’ views varied around three issues: information provided, professionals’ behaviours and the aim of persuasion.

MHPs described the aim of persuasion as being a significant discriminant between it being perceived as a non-coercive or coercive influence strategy. Some MHPs defined persuasion as a non-coercive strategy when a patient is presented with the therapeutic alternative as a sequence of choices expressed by the inclusive disjunction “and/or” (E3 Motivation). Other MHPs formulated persuasion using a conditionality that simplified the therapeutic offer to two options, generally opposed and expressed by the exclusive disjunction “or/or” (E7 Threat). Participants saw the former as a strategy to promote free choice and the latter as a conditional offer, where the patient can choose only between two available options.

MHPs’ behaviour was another distinguishing factor. Some participants considered persuasion a strategy to assess patients and to help them make the best choice and prevent harm (E2 Assessment). For other MHPs, persuasion was essentially seen as a strategy to convince the patient during negotiations about treatment (E5 Deceit), using specific professional skills to “seduce” and “hook” the patient (E6 Manipulation). This latter use of persuasion has been frequently associated with a paternalistic attitude that justifies the use of information as a lever to improve compliance in the patient’s best interests (E4 Informational manipulation).

Finally, the type of information provided and how the information is delivered were also considered potential factors for differentiating between the “non-coercive” and “coercive” use of persuasion. Some participants defined information as a process helping patients to become aware of their clinical circumstances (E1 Professional information). According to their views, persuasion appeals to patients’ rationality. It provides them with relevant content to support their choice of therapeutic options, as well as other information related to side effects, treatment consequences, risks, benefits and patient rights. Other participants described persuasion as informational manipulation consisting of withholding or laying out specific information to either emphasise or minimise risks or to justify what was previously in the professional’s mind (E8 Conditional offer).

### Interpersonal leverage: emotional handling and affective manipulation

Quotes represented in Table 4 describe how Interpersonal leverage involves influencing patients’ choices by exerting leverage on their affective and emotional reactions (E10 Handling emotions). This strategy can be applied successfully in an extended and well-consolidated user–provider relationship or when it involves other members of the patient’s social network, such as family members, friends, case managers, GPs or whatever MHP plays a relevant, influential role in the patient’s life (E9 Social network involvement). MHPs’ views about the use of interpersonal leverage were heterogeneous and conflicting. Some participants considered handling a patient’s emotions was appropriate when it was conducted in the best interests of the patient and always oriented to improve a patient’s respect for MHPs (E11 Respectful reciprocity) or to increase a patient’s confidence in the community services (E12 Confidence)*.* In contrast, other participants associated interpersonal leverage with manipulating a patient’s emotional life when the trusting relationship between the patient and social network is used as a lever of confidence to drive the patient’s choices (E13 Leverage on confidence). This strategy is considered to be harmful and abusive to the therapeutic alliance because it undermines patient trust. For that reason, some participants defined interpersonal leverage as coercive and unprofessional (E14 Unprofessional) and clinically wrong (E15 Wrong).

### Inducement: incentives as a lure and conditional offer

Table 5 quotes describe how, inducement fosters compliance using incentives, which are conditioning factors operating on a patient’s choice. Factors that influenced MHPs’ perceptions were using incentives as a lever, the communication style and the patient’s awareness of choice.

Most participants agreed that inducement implies conditionality and is different from other pressures commonly used in community care. However, the findings indicated discrepancies with regard to how inducements are applied and their effect on the user-provider relationship. Whether an inducement was perceived as non-coercive or coercive depended on the type of incentive used to entice the patient. Non-coercive inducements retained compensation for patients to meet their obligations and complete the therapeutic contract (E17 Negotiation). In contrast, coercive inducements represented a loss of some existing benefits (e.g. financial incentives, housing or other community benefits; E19 Enticement). Under these circumstances, rather than a therapeutic contract, the inducement was defined as “blackmail” and “manipulation” (E21 Manipulation), with a dubious therapeutic efficacy (E22 Harm user–provider relationship).

Communication style also played a role in MHPs’ views about how inducements are expressed. MHPs believed that positive conditionality is expressed using the inclusive disjunction “and/or”, whereas negative conditionality is more frequently associated with the exclusive disjunction “or/or” (E16 Positive and negative reinforcement). MHPs who defined inducement as a positive offer thought that incentives are very effective in improving patient awareness of treatment options and the resources available to make a free choice (E18 Therapeutic agreement). Alternatively, MHPs who identified inducements as “coercive” conditional offers were aware of the undeniable coercive nature of the inducements (E20 Coercion) but justified their use when trying to make decisions in a patient’s best interests (E23 Extortion in the patient’s best interests).

### Threat: realistic and biased information

The threat is depicted in Table 6 as an influence strategy that aims to modify a patient’s behaviour by withholding some benefit or resulting in the loss of freedom due to involuntary hospitalisation or the involvement of the legal system. Participants’ views about the use of threat converged on the intrinsic conditionality of this lever, consisting of a therapeutic offer focused only on the consequences of decision making and expressed through disjunctive language oriented to present consequences as the only two options available and to undermine the patient’s choice.

As with other influence strategies, threats assumed different values depending on how MHPs used information. Disagreements and different views about the meaning and use of information when using threats affected participants’ perceptions of their therapeutic value and their effects on the user–provider relationship and patient well-being (E29 Highest level of coercion). Some participants described a threat strategy as unacceptable under medical deontology for ethical, legal and professional reasons. One participant referred to the use of threats as an act against professional guidance and deserving of sanctioning by the College of Physicians (E30 Unethical). The principal argument used to support this view was the harm caused to the user–provider relationship, the loss of trust and the related deterioration in the therapeutic alliance (E31 Untrustworthy).

Other MHPs offered a different view about the threat strategy. Even though they recognised the coercive power of this strategy, they depicted threat as a rhetorical strategy centred on the impact of the language and the arguments provided (E28 Communication strategy). The threat was presented as a frank reassessment of the possible consequences of choices based on the current clinical circumstances (E24 Reality Assessment).

These views highlight the realism and trueness of the information regarding the consequences provided to the patient more than the potentially harmful effect on patient autonomy (E25 Factual information). The reality of the events and their evocative power is the most significant features of the information provided in the threat (E26 Actual threat or anticipation). The trueness of the information reduces the threat to a risk–benefit balance and indicates to patients the destructive power of an imminent reality if they refuse to comply with the professionals’ recommendations (E24 Risk–benefit balance).

### Influences strategies: commonalities and differences

We created a logic model of the different influence strategies examined in this study (Table 7), summarising commonalities and differences and how participants described each of the strategies as coercive (leveraged) or non-coercive (non-leveraged). Three features characterised participants’ descriptions: using a lever, making a conditional or biconditional offer, and leading patients towards a free or controlled choice. Influences may be leveraged or not; if they are leveraged, they are seen as manipulative (E4; E6; E13; E21); if they are not, they are seen as a form of communication (E3; E11; E18; E25). Manipulation may assume different shapes (e.g. emotional blackmail (E5), seduction (E4), and reverted psychology (E6). Communication can take the form of motivation (E3), engagement (E11), a therapeutic contract (E18), professional information (E1) and assessment (E2; E24).

**Table 7:**
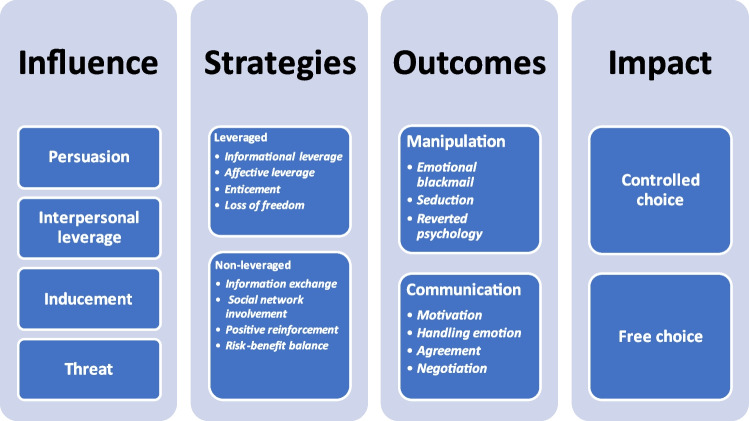
Logic model showing the use of influence in mental health community care depicted by the study participants

Participants described conditionality as biconditional if it involved two mutually exclusive options that the patient is forced to choose between (E7; E8; E16; E19, E21). Under these circumstances, the choice results from MHPs’ views about the treatment and what they consider is in the patient’s best interests (E10; E20; E23; E31). The level of control exerted on a patient’s choice is seen as a reduction in freedom because the focus is only on the consequences of decision-making (E5; E13; E16; E28). However, non-coercive influences promote informed (E2) and rational (E17) choices supported by unbiased information (E3; E25) and patient awareness of the choice (E18). MHPs’ communication style is characterised by conditional statements about therapeutic options that the MHPs advise are provided in terms of a possible free choice (E3; E17; E27).

## Discussion

### Main findings

In the present study, we used empirical bioethics analysis to explore two aspects that were missed in the primary data analysis [29], namely the normative consequences associated with the description of influence provided in the literature and the ethical theories about coercion. Our findings indicate that the difference between persuasion and coercion is controversial and that MHPs have different views about what “informal coercion” is. These differences are due not only to differences in clinical practice and health care systems but also to how ethical theories from empirical research are interpreted and used to explain or describe the medical practice.

Empirical bioethics combines the need to explain what duties are and how they are related to what people do. To extract a normative conclusion that can help MHPs overcome the barriers related to the use of influence [3, 30–33] we have used a consultative approach to balance the theory about informal coercion and MHPs’ descriptions of their experience. Our analysis collated all the aspects that contributed to engendering the different views identified among participants and suggested some normative conclusions inherent to their practice. In accordance with the primary study [29] and a new international qualitative study [16], the present secondary analysis identified several commonalities between the institutions involved in the study. Despite the diversity in healthcare systems, this study found that MHPs’ had comparable views about the difference between persuasion and informal coercion.

Empirical research in mental health has depicted influence strategies using two different descriptions, one proposed by Carrol [39], who referred to force, manipulation and persuasion, and the other proposed by Wertheimer [20], who talked about coercion, inducement, persuasion and authority. Such interpretations have drawn on two different theories of coercion, termed, by Anderson [40], as the “enforcement approach” and the “pressure approach”. The former focuses on the role of the coercer and defines coercion as the coercer's power to modify the coercee’s behaviour. The latter stresses the role of the coercee and depicts coercion as a manipulative strategy operating on the coercee’s will [41]. The “enforcement approach” comes from political philosophy and defines coercion as power [42], whereas the “pressure approach” comes from law and analytical philosophy and conceptualises coercion as a threat [43, 44]. Because Carrol’s and Wertheimer’s descriptions [29, 30] of influence have prevailed in the mental health literature, empirical research has focused on the roles of MHPs and patient perceptions. Our findings suggest that levers and the type of leverage used in communications with the patient are also relevant to differentiating persuasion from coercion. Furthermore, levers are crucial to understanding the difference between leveraged and non-leveraged influence, and the normative consequence from this insight is vital.

The description of treatment pressures as a hierarchy of influences with different degrees of intensity, ranging from the simple advice provided in psychotherapy to the use of restraints and seclusion, has a normative value because it makes a moral distinction between threat, the only pressure properly defined as coercion [21], and the other types of leverage. Our findings indicate that conditionality is not only a characteristic of inducement and threat, but may also be attributed to every influence strategy when using a lever and converting a patient’s decision-making into a conditional choice.

Szmulker and Appelbaum [21] synthesised the distinction between positive (persuasion and inducement) and negative (threat and force) pressures [4, 6, 24, 45] with the concept of a continuum expounded by the social control theory [46–49] which describes social control as a spectrum from coercion to voluntarism [47], differentiated according to which influences are invisible or covert: coercion, coerced voluntarism (threat), utilitarian compliance (inducement) and persuasion [49]. This original conceptualisation of influence does not make any normative difference between the different types of influence; they are all equal forms of social control, more or less visible. Combining the concept of a continuum with Wertheimer’s [20] moral baseline argument makes a moral differentiation of influence possible. Empirical research assigning such distinctions a normative value enables differences between pressures and levers to be established: persuasion and inducement are positive because, if the patient accepts them, he will be better off; however, threat and force are negative because, if the patient accepts them, he will be worse off [24]. Our findings indicate no qualitative difference and a corresponding lack of normative significance between influences because all are strategies to exert control over the patient. What does differ is how this control is exercised and whether the influences are leveraged or not, and how this determines the patient’s choice. The main finding of our secondary analysis is the need to rethink the idea that the more intense the influence, the greater the moral justification required [11]. All levers require moral justification, and this is only possible if MHPs are aware of what a lever is and how it affects a patient’s choice.

### Comparison with the literature

Levers in mental health community care have been defined as a means to control the behaviour of voluntary patients and promote compliance [50, 51]. In this study, we identified three essential types of levers, namely those acting on a patient’s affectivity and emotional life, those related to the information provided to the patient and the use of incentives.

The first type of levers can be defined as emotional because they are applied through the affective power exerted by the social network on patients [52]. Emotional levers play a significant role in patient adherence [6, 29–31]. However, they may significantly increase a patient’s emotional dependency on carers [21] and contribute to patient disengagement [53].

The second type of levers, informational levers, are applied by choosing which information to provide in order to persuade another to change an attitude or behaviour [6]. For example, withholding or providing select information to induce a change in a patient’s behaviour is termed “informational manipulation” [54]. The aim of informational leverage is to develop a cognitive bias that influences patients’ decision-making. This cognitive bias is the mistaken belief produced as a result of providing select information that is not known by the patient but is developed ad hoc by the informant [55]; for example, talking about the risk of treatment by referring only to the relative rather than absolute risk is a way of manipulating the patient [56].

The third type of levers are incentives, such as money, housing and hospitalisation [57, 58]. Financial incentives may involve a patient’s relative or a member of their social network (e.g. a payee named by social services to control a patient’s financial resources) [6]. Housing is frequently used as a lever for hospital discharge and is considered effective because it reduces hospitalisation and enhances participation in productive life activities [59]. However, housing as a lever has been severely criticised and considered unethical because it denies the patient’s civil right to have a house [60]. Finally, levers may also consist of legal measures where specific treatment conditions are linked to a patient’s conditional release on probation or parole and are used in mandated community treatment [45, 61]. Despite the frequent use of levers in community care [61, 62], their impact on patient satisfaction is underestimated [30].

### Conditionality

The findings of this study describe how the use of levers has a significant impact on the meaning of a therapeutic offer and how MHPs associate that with persuasion or coercion. Participants defined leveraged influence as conditional offers and described conditionality as a beneficial aspect for understanding the function of levers. Data depicted conditionality as a particular communication style corresponding to non-leveraged and leveraged influence. Using a formal logic conceptualisation, we refer to the former as a conditional statement (if *p*, then *q*) and the latter as a biconditional statement (*p* if and only if *q*). Conditionality is a controversial subject; some philosophers identify conditionality with coercion and define coercion as a threat (e.g. when the threatener will act against the threatened regardless of other contingencies). A successful conditional proposal presumes a background relationship of power between the coercer and coercee, which provides the coercer’s threat enough power to modify the coercee’s behaviour [41]. However, other philosophers have identified conditionality as coercion only in circumstances where conditional proposals are biconditional in nature [63]. Indeed, this perspective defines conditional proposals as constituted by two conjuncts, one representing a positive case and the other representing a negative case; positive or negative consequences follow in reason to the case accepted by the person making the choice. When the second conjunct of the statement expresses a lack of result concerning what is described in the first conjunct (e.g. when the proposer does nothing as a consequence of what the chooser does), the statement loses its coercive power, leaving other options available regarding the only option indicated in the conditional statement [64]. Conditionality is not necessarily coercive, but can be coercive in exceptional circumstances, i.e. when a proposal is a biconditional.

Both meanings of conditionality have been reported in the mental health literature. In some studies, conditionality has a more general purpose and describes those influence strategies where MHPs try to convince the patient to accept a therapeutic offer [65] or a therapeutic agreement [45]. In other studies, conditionality is also depicted by patients and MHPs as manipulation [61] or blackmail [28]. However, in some cases, the term is more detailed, and the distinction between conditional and biconditional is used to associate informal coercion only with biconditionality. For example, Szmulker and Appelbaum [21] attributed conditionality only to the inducement and threat because both are expressed through biconditional propositions. Conditionality is described as a characteristic of levers by Burns et al. [61], who distinguished non-leveraged pressures as non-conditional because they are not matched with direct consequences following the patient’s choice.

### Communication and manipulation: rational choice and control

Even though conditionality is defined in the mental health literature referring to both conditional and biconditional statements, what is relevant to understanding our data is the relationship between that concept and the type of choice. The findings show two different types of choice, one depending on what MHPs have in mind and their ideas about the aims and consequences of the therapeutic offer and the other built upon a patient’s awareness of their clinical circumstances. In the case of the former type of choice, the provider–client relationship is not based on trust and motivation but rather on manipulation and deceit and is in the patient's best interests. The latter is an informative process whereby MHPs provide an assessment focused on reciprocity and confidence and aim to define a therapeutic agreement. This conceptual distinction between conditionality and biconditionality describes patients’ choices and how they may be affected by the use of levers. When influences are leveraged, patients’ freedoms are unavoidably reduced, and decision-making does not occur due to rational and careful information processing. Still, the information is conditioned by selecting the content and how it is delivered. Manipulation operates on a patient’s motivation and ability to process information objectively and enhances or reduces understanding [66]. Freedom and control have different natures; the former comes from the opportunity to choose, whereas the latter comes from making a concrete choice in a specific context. “Freedom” refers to the absence of external causation; “control” refers to internal–intentional causation. In the case of uncontrollable external events, a patient can lack both freedom from these events and control over them [39]. Levers and conditionality (or biconditionality) constitute the circumstances of a patient’s choice control, and informal coercion represents the lack of opportunities to choose among alternative courses of action because the coercer has removed one or more options, has made them less desirable or has reduced the coercee’s ability to choose and act.

### Strengths and limitations

The model of informal coercion used to define influence strategies in this study is consistent with that frequently used in the literature. Furthermore, the focus groups were conducted in national languages by multilingual facilitators who were also involved in the analysis. The primary study was conducted by a multidisciplinary team (ethicists, psychiatrists, psychologists, and social workers). However, our findings need to be considered in light of some study limitations. First, this study was conducted with clinicians who had different qualifications and worked in countries with very different cultures. The lack of patient participation in the discussion may represent a significant limitation, resulting in a one-sided description of influences and their impacts on the quality of care. However, others have reported patient descriptions of how influence is used not only to improve compliance, but also to improve adherence to social norms and how this depends on the institutional setting [17]. Second, the health systems of the countries involved in this project include various mental health services providing outpatient care. It is widely recognised that certain characteristics of mental health systems in different countries can lead to different levels of experienced coercion, as suggested by Molodinsky et al. [22]. However, our analysis did not address how country-level characteristics of mental health systems may be linked to differences in the impact of influence. Third, the sites included in the study were chosen on the basis of the network of the researchers leading the study. Fourth, the analysis presented in this paper is secondary because the primary research question was on informal coercion in general. However, the definitions of persuasion and leverage were explained to participants in each of the 36 focus groups, and appropriate time was allocated for discussion. Fifth, during the analysis, a saturation grid was developed following the recommendations of Brod [67]. Saturation appeared to have been reached on the specific questions at the national level (four focus groups) and for comparisons of all countries (36 focus groups). Sixth, some specific language nuances may have been lost during transcript translation into English, but this is unlikely to have affected the general themes and characteristics inferred from the available material. Finally, the focus groups were conducted in 2014 as part of the primary study. However, the theoretical model discussed in this secondary analysis considers the most recent investigations and conceptualisations about the subject [31–33].

## Conclusion

In this study, we identified characteristics within the range of influence strategies used in community care that differentiate “non-coercive” from “coercive” influence strategies. The latter is characterised by using a lever, expressing clinical decisions as conditional offers and not allowing patients free choice about therapeutic proposals. Our findings provide empirical validation and specification in the practice of theoretical frameworks on coercion in mental health care. The characteristics differentiating “non-coercive” and “coercive” influence strategies can be used to facilitate and standardise reflection on influence strategies in community mental health care. This could help reduce the diversity of practice and, in particular, avoid unintended coercive practices. Further research should test the use of these overarching principles when discussing and reflecting on influence strategies in individual supervision and reflective groups, and their impact on both professionals’ experiences and patients’ perception of care.

## Data Availability

The dataset used in this study will not be shared due to ethical requirements established by the research ethics committee in Spain for the entire study and, at the national level, by the other research ethics committees involved in the ethics assessment of the protocol. Anonymised data will be available upon reasonable request to the corresponding author.

## Abbreviation

MHPs: Mental health professionals
